# Antitumoral Action of Resveratrol Through Adenosinergic Signaling in C6 Glioma Cells

**DOI:** 10.3389/fnins.2021.702817

**Published:** 2021-09-01

**Authors:** Alejandro Sánchez-Melgar, Sonia Muñoz-López, José Luis Albasanz, Mairena Martín

**Affiliations:** Department of Inorganic, Organic Chemistry and Biochemistry, Faculty of Chemical and Technological Sciences, School of Medicine of Ciudad Real, Regional Center of Biomedical Research (CRIB), Universidad de Castilla-La Mancha, Ciudad Real, Spain

**Keywords:** resveratrol, glioma, CD73, adenosine receptor, brain

## Abstract

Gliomas are the most common and aggressive primary tumors in the central nervous system. The nucleoside adenosine is considered to be one major constituent within the tumor microenvironment. The adenosine level mainly depends on two enzymatic activities: 5′-nucleotidase (5′NT or CD73) that synthesizes adenosine from AMP, and adenosine deaminase (ADA) that converts adenosine into inosine. Adenosine activates specific G-protein coupled receptors named A_1_, A_2A_, A_2B_, and A_3_ receptors. Resveratrol, a natural polyphenol present in grapes, peanuts, and berries, shows several healthy effects, including protection against cardiovascular, endocrine, and neurodegenerative diseases and cancer. However, the molecular mechanisms of resveratrol actions are not well known. Recently, we demonstrated that resveratrol acts as an agonist for adenosine receptors in rat C6 glioma cells. The present work aimed to investigate the involvement of adenosine metabolism and adenosine receptors in the molecular mechanisms underlying the antitumoral action of resveratrol. Results presented herein show that resveratrol was able to decrease cell numbers and viability and to reduce CD73 and ADA activities, leading to the increase of extracellular adenosine levels. Some resveratrol effects were reduced by the blockade of A_1_ or A_3_ receptors by DPCPX or MRS1220, respectively. These results suggest that reduced CD73 activity located in the plasma membrane in addition to a fine-tuned modulatory role of adenosine receptors could be involved, at least in part, in the antiproliferative action of resveratrol in C6 glioma cells.

## Introduction

Gliomas are the most common primary tumors of the central nervous system (Wesseling and Capper, 2018). These types of brain tumors have particularly aggressive behavior with a high recurrence rate. Although the current therapeutic approach combines surgical intervention, irradiation, and adjuvant chemotherapy, the prognosis is still very poor for these tumors. Thus, there is a need to find new strategies to improve glioma treatment and reduce its recurrence rate. Adenosine is a key mediator of several biological functions involving multiple signaling pathways (Borea et al., 2018) and mainly operates through four G-protein coupled receptors named A_1_, A_2A_, A_2B_, and A_3_. Adenosine A_1_ and A_3_ receptors are coupled to Gi/o-proteins and inhibit adenylyl cyclase activity. In turn, adenosine A_2A_ and A_2B_ receptors are coupled to Gs-proteins and stimulate adenylyl cyclase activity. Adenosine can be formed intracellularly and exported *via* transporters or extracellularly with the participation of CD73 activity as an adenosine-generating enzyme from adenine nucleotides, as ATP, released from cells. Adenosine can be transformed into inosine by adenosine deaminase activity (Fredholm et al., 2011). Because adenosine is considered one of the major constituents within the tumor microenvironment (Di Virgilio and Adinolfi, 2017), adenosinergic signaling has emerged as a potential therapeutic strategy in cancer (Allard et al., 2012). However, the precise procedure to target adenosine-mediated signaling remains under discussion as two different tissues may be affected, the tumoral cells and the immune system. Adenosine seems to be involved in tumor generation, growth, invasion, angiogenesis, and metastasis through activation of all four adenosine receptor subtypes (Gessi et al., 2011; Allard et al., 2012). Nevertheless, adenosine appears to have an opposite biological action as tumor-derived adenosine induces A_2A_ receptor activation from immune cells, leading to an immunosuppressive state of the immune system and, thus, facilitating tumor growth (Ohta et al., 2006). In addition, CD73 is suggested as a key enzyme in tumor growth (Zhang, 2012; Yan et al., 2019). In agreement, its inhibition or depletion causes cell growth inhibition in different *in vitro* (Bavaresco et al., 2008; Zhu et al., 2017) and *in vivo* models (Stagg et al., 2011, 2012). In fact, several drugs and antibodies targeting CD73 are under study in clinical trials due to their potential role in cancer (Buisseret et al., 2018).

Resveratrol (RSV) is a polyphenolic compound present in plants, such as peanuts and grapes, and it shows multiple healthy properties in several diseases, including cancer (Carter et al., 2014; Jiang et al., 2017; Ko et al., 2017). This phytochemical emerged as a promising molecule since the first time its effectiveness was reported against cancer in both *in vitro* and *in vivo* models (Jang et al., 1997; Kiskova et al., 2020). Unfortunately, the action mechanism by which this polyphenol exerts its antitumoral activity remains not well understood. Recently, we reported that RSV acts as a non-selective agonist for adenosine receptors in rat C6 glioma cells (Sanchez-Melgar et al., 2019). Moreover, RSV induces *in vivo* changes in adenosinergic signaling by modulating the functionality of A_1_ and A_2A_ receptors in the brain from SAMP8 mice after long-term RSV supplementation in their diet (Sanchez-Melgar et al., 2018). Therefore, the aim of the present work was to investigate whether RSV treatment is able to modulate adenosine-converting enzymes and whether adenosinergic signaling is somehow involved in the antitumoral action of this polyphenol in C6 glioma cells.

## Materials and Methods

### Chemicals

*Trans-*RSV (ref. R5010), caffeine (CAF) (ref. C-0750), N^6^-cyclopentyladenosine (CPA) (ref. C-8031), 4-(3-Butoxy-4-methoxybenzyl)-2-imidazolidinone (Ro-20-1724) (ref. 557502), and adenosine 5′-triphosphate disodium salt hydrate (ATP) (ref. A7699) were purchased from Sigma Aldrich; 2-[p-(2-carboxyethyl) phenylamino]-5′*-N-*ethylcarboxamido adenosine (CGS21680) (ref. 1063), *N-*[9-Chloro-2-(2-furanyl)[1,2,4]-triazolo[1,5-c]quinazolin-5-yl]benzene acetamide (MRS1220) (ref. 1217), 1-[2-Chloro-6-[[(3-iodophenyl)methyl]amino]-9H-purin-9-yl]-1-deoxy-*N*-methyl-β-D-ribofuranuronamide (2- Cl-IB-MECA) (ref. 1104), 4-(2,3,6,7-Tetrahydro-2,6-dioxo-1-propyl-1H-purin-8-yl)-benzenesulfonic acid (PSB1115) (ref. 2009), 8-Cyclopentyl-1,3-dipropylxanthine (DPCPX) (ref. 0439), 2-[[6-Amino-3,5-dicyano-4-[4-(cyclopropylmethoxy)phenyl]-2-pyridinyl]thio]-acetamide (BAY606583) (ref. 4472), 4-(2-[7-Amino-2-(2-furyl)[1,2,4]triazolo[2,3-*a*][1,3,5]triazin-5- ylamino]ethyl)phenol (ZM241385) (ref. 1036), and 2′(3′)-O-(4-Benzoylbenzoyl)adenosine-5′-triphosphate tri(triethyla mmonium) salt (BzATP) (ref. 3312) were purchased from Tocris. Calf intestine adenosine deaminase (ADA) (ref. 10102121001) was purchased from Roche. Other used reagents are indicated in their corresponding section.

### Cell Culture

Rat C6 glioma cells were obtained from the American Type Culture Collection (ref-CCL-107) and grown (passages 40–60) in Dulbecco’s modified Eagle’s medium (DMEM) supplemented with 10% fetal bovine serum, 2 mM L-glutamine, and 1% non-essential amino acids and antibiotics in a humidified atmosphere of 95% air, 5% CO_2_ at 37°C. As the potency of adenosinergic ligands depends on the model, species (rat, human, or mouse), tissue, and overall experimental conditions of the assay (Fredholm et al., 2011), C6 cells were subjected to different adenosinergic ligands at concentrations that were selected considering the pharmacological characterization of adenosine receptors (Castillo et al., 2007) and the characterization of RSV as an adenosine receptor agonist in these cells (Sanchez-Melgar et al., 2019). For instance, PSB1115 and CGS21680 used in the range 0.1 nM–1 mM were unable to displace binding of 20 nM [^3^H]DPCPX to A_1_ receptors in intact C6 cells. Similarly, PSB1115 and CPA used in the range 0.1 nM–1 mM were unable to displace binding of 15 nM [^3^H]ZM241385 to A_2A_ receptors in intact cells (Castillo et al., 2007).

### Cell Viability Assays and Cell Counting

Cells were plated in 96-well dishes (10^4^ cells/well) and grown overnight before starting treatment. After treatment, cell viability was assessed by the XTT method following the manufacturer’s instructions (Roche). Reagents were incubated for 150 min at 37°C, and absorbance was measured at 475 and 690 nm on a Synergy HT (BIO-TEK) plate reader. The results are expressed as percentages relative to the control condition. Cells grown in six-well dishes (5⋅10^5^ cells/well) were detached and counted on a TC 10^TM^ Automated Cell Counter (BioRad) after treatment and compared with the corresponding controls.

### Caspase-3 Activity

Cells from each condition (10^6^ cells) were used as indicated by the manufacturer’s protocol (Molecular Probes, Barcelona, Spain). Cells were lysed for 30 min at 4°C and centrifuged at 12,000 rpm for 5 min. Supernatant (50 μL) was collected, and a mix containing Z-DEVD, DTT, EDTA, PIPES, and CHAPS was added into the P96-black well. After 30 min of incubation at room temperature protected from light, fluorescence was read at Ex/Em of 340/440 nm, respectively, in a kinetic mode for 4 h. Slope value was used to represent the enzymatic activity. Samples from each condition were analyzed in duplicate at the same cell passage.

### Cell Cycle Assays

After treatment, cells were washed with phosphate buffer (PB) and incubated with trypsin to detach cells. After trypsinization, cells were centrifuged, and the obtained pellet was carefully resuspended in 100 μL PB. Cells were then fixed by adding cold ethanol for 4 h at 4°C. After fixation, cells were washed to remove ethanol and staining solution containing 0.1% Triton X-100, 10 μg/ml of propidium iodide (Molecular Probes, Inc.), and 50 μg/ml RNase A in PB was added; samples were incubated for 30 min at room temperature. Fluorescence was detected at 488 nm on a MACSQuant^®^ 10 flow cytometer.

### Nuclei Staining

Cell nuclei were visualized by fluorescence microscopy using Hoechst 33258 (Sigma-Aldrich, Madrid, Spain) as a staining method. Briefly, culture media was removed, and cells were washed with PBS (pH 7.4). Cells were fixed with 4% paraformaldehyde for 10 min at room temperature. After washing three times for 10 min in PBS, nuclei were stained with 1 μg/ml Hoechst for 10 min protected from light and mounted with ProLong Gold antifade reagent (Invitrogen, Madrid, Spain). Nuclei were quantified using a DMI6000B microscope and LAS AF software (Leica Microsystems, Wetzlar, Germany).

### Plasma Membrane Isolation

Plasma membrane isolation was performed as previously described (Luis Albasanz et al., 2002). Cells were homogenized in isolation buffer (50 mM Tris–HCl, pH 7.4, containing 10 mM MgCl_2_ and protease inhibitors) with Dounce homogenizer (10XA, 10XB). After homogenization, samples were centrifuged for 5 min at 1,000 × *g* in a Beckman JA 21 centrifuge. Supernatants were centrifuged again for 30 min at 27,000 × *g*, and the resulting supernatant was considered to be the cytoplasmic fraction, and the pellet (plasma membrane) was resuspended in isolation buffer. Protein concentration was measured by the Lowry method.

### 5′-Nucleotidase Activity

Isolated plasma membrane (20 μg) and cytosolic (20 μg) fractions were preincubated in 180 μL of the reaction medium containing 50 mM Tris, MgCl_2_ 5 mM, pH 9, at 37°C for 10 min. Then, the reaction was initiated by the addition of 20 μL AMP at a final concentration of 500 μM and stopped 20 min later by adding 200 μL of 10% trichloroacetic acid. The samples were chilled on ice for 10 min and then centrifuged at 12,000 × *g* for 4 min at 4°C. The supernatants were used to measure the inorganic phosphate released following the protocol described earlier (Leon-Navarro et al., 2015) using KH_2_PO_4_ as Pi standard. Non-enzymatic hydrolysis of AMP was used as a blank. Incubation times and protein concentration were selected to ensure the linearity of the reactions. All samples were run in duplicate. 5′-Nucleotidase (CD73) activity is expressed as nmol Pi released/min/mg of protein.

### Adenosine Deaminase Activity

An adenosine deaminase (ADA) activity assay (ref. ab204695) was performed in a 96-well plate according to the manufacturer’s protocol (Abcam, Cambridge, United Kingdom). The cytoplasmic fraction was diluted 1:100 in ADA buffer assay and assayed in duplicate. Then, the 96-well plate was read at Ex/Em = 535/587 nm as a kinetic curve for 30 min. ADA activity was obtained by interpolation in an inosine standard curve performed in parallel in the same plate. Enzymatic activity was normalized to the amount of protein.

### Adenosine and Related Metabolite Detection by HPLC

Chromatographic analysis was performed with Ultimate 3000U-HPLC, and data peaks were processed with Chromeleon 7 (ThermoFisher, Madrid, Spain) as previously described (Alonso-Andres et al., 2019). An HPLC diode array was used working at 254 nm wavelength. Purine standards and samples (40 μL) were injected into a C18 column of 4.6 × 250 mm, 5 μm particle size. Two solvents were used for gradient elution: solvent A, 20 mM phosphate-buffered solution (pH 5.7), and solvent B, 100% methanol. The gradient was 95% (11 min), 80% (9 min), and 95% (2 min) in solvent A. The total run time was 22 min with a constant flow rate of 0.8 mL/min at 25°C. Retention times for inosine and adenosine were 8.4 and 15.5 min, respectively. Each purine level was obtained by interpolation from the corresponding purine standard curve. The standard curves were obtained by using five concentrations of each purine ranging from 0.1 to 500 μM. Data were then normalized to the protein concentration of each sample.

### Cell Microscopy and Population Doubling Time Calculation

C6 glioma cell growth was recorded with a digital camera (Leica DFC350FX) attached to a Leica DMI6000B (Leica Microsystems, Wetzlar, Germany) fluorescent microscope using × 20 HCX PL FLUOTAR objective. Cells were maintained at 5% CO_2_ and 37°C in a stage-top incubation system (PeCon GmbH, Erbach, Germany) during video recording (one image every 2 min). Cells were counted every 3 h, and the relative increase respect time 0 was calculated. Population doubling time was obtained from the fold increase data by non-linear regression fitting curve to exponential growth equation.

### Statistical and Data Analysis

Statistical analysis was according to Student’s *t*-test. Differences between mean values were considered statistically significant at *p* < 0.05. GraphPad Prism 6.0 program was used for statistical and data analysis (GraphPad Software, San Diego, CA, United States). Cell cycle histograms were analyzed with FlowLogic 7.3 software by Inivai Technologies (Victoria, Australia).

## Results

### Effect of RSV and the Pharmacological Stimulation/Blockade of Adenosine Receptors in C6 Glioma Cell Growth and Viability

**FIGURE 1:**
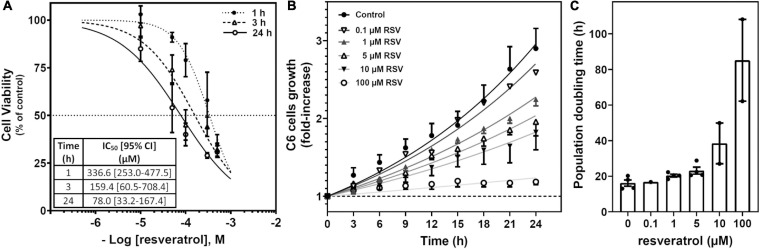
Effect of RSV on C6 glioma cell growth. **(A)** Cell viability based on the XTT method was performed after RSV exposure for 1, 3, and 24 h at different concentrations. Data are means ± SEM of three to six independent assays. **(B)** Cells were treated with different concentrations of RSV as indicated. Cells were counted from phase-contrast images recorded (one frame every 2 min) for 24 h at the indicated interval and relativized to the number of cells at the beginning of treatment. A representative video can be seen in Supplementary Video 1. **(C)** Population doubling time derived from three to five video recordings as represented in panel **(B)**.

Cells were treated with RSV at different times and concentrations to assess the ability of this polyphenol to cause cell death. Cell viability assays were performed based on the XTT reduction method, and the antitumoral activity of RSV was analyzed. Cell viability was diminished in a time- and concentration-dependent manner (Figure 1A), revealing that treatment at 100 μM RSV for 24 h reduced about 50% of the measurement of cell viability. The proliferation of cells was gradually decreased as RSV concentration was increased from 0.1 to 100 μM (Figure 1B and Supplementary Video 1), and the population doubling time (Figure 1C) changed from 16.1 ± 1.7 h in control cells to 85.1 ± 22.9 h in the 100 μM RSV treatment. This concentration and time of treatment were used for the next set of experiments with RSV.

**FIGURE 2:**
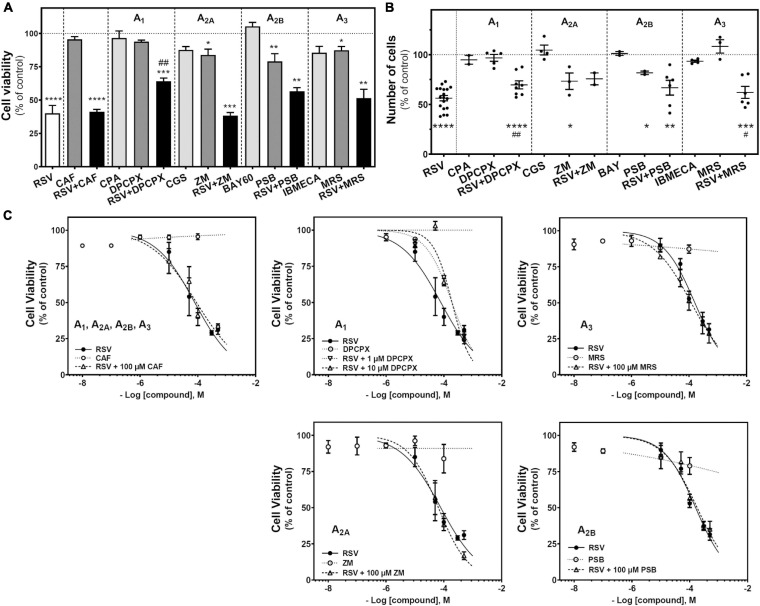
Effect of RSV and adenosinergic ligands on C6 glioma cell growth. **(A)** Cell viability after 24 h of treatment with 100 μM RSV, 100 μM CAF, 10 μM CPA, 10 μM DPCPX, 10 μM CGS, 100 μM ZM, 10 μM BAY60, 100 μM PSB, 10 μM IBMECA, and 10 μM MRS alone or in combination. **(B)** Number of cells after 100 μM RSV, 10 μM CPA, 10 μM DPCPX, 10 μM CGS, 100 μM ZM, 10 μM BAY60, 100 μM PSB, 10 μM IBMECA, and 10 μM MRS alone or in combination after 24 h of treatment. **(C)** Cell viability after 24 h treatment with different concentrations of RSV, CAF, DPCPX, ZM, PSB, and MRS alone or in combination. Data are means ± SEM of 3–10 independent assays. *p < 0.05, **p < 0.01, ***p < 0.001 and ****p < 0.0001 significantly different from control condition according to Student’s *t*-test. #*p* < 0.05 and ##*p* < 0.01 significantly different from RSV condition. RSV, resveratrol; CAF, caffeine; CPA, N^6^-cyclopentyladenosine; DPCPX, 8-Cyclopentyl-1,3-dipropylxanthine; CGS, CGS21680; ZM, ZM241385; BAY60, BAY606583; PSB, PSB1115; IBMECA, 2-Cl-IB-MECA; MRS, MRS1220.

Resveratrol is a non-selective agonist for adenosine receptors, showing a strong influence in A_2A_-mediated signaling (i.e., G protein coupling switch from Gs to Gi) after acute RSV treatment at high concentrations. Therefore, we target adenosine receptors with selective agonists and antagonists (10 μM CPA and 1 and 10 μM DPCX for A_1_ receptors; 10 μM CGS21680 and 100 μM ZM241385 for A_2A_ receptors; 10 μM BAY606583 and 100 μM PSB1115 for A_2B_ receptors; 10 μM IBMECA and 10 μM MRS1220 for A_3_ receptors) to investigate the role that these receptors play on C6 glioma cell growth. Caffeine (100 μM), a non-selective antagonist for adenosine receptors, was also employed. As Figure 2A shows, the activation of A_1_ (CPA), A_2A_ (CGS21680), A_2B_ (BAY606583), or A_3_ (IBMECA) receptors did not induce significant changes in cell viability after 24 h of treatment. In turn, the blockade of A_2A_ (ZM241385), A_2B_ (PSB1115), or A_3_ (MRS1220) receptors significantly reduced the cell viability. Similar results were obtained when the number of cells after treatment was analyzed (Figure 2B). The blockade of the A_2A_ receptor significantly reduced the number of cells. Interestingly, blockade of A_1_ receptor with DPCPX during RSV treatment significantly reduced the RSV effect on cell viability (Figures 2A,C) and the number of cells (Figure 2B). In turn, the blockade of the A_3_ receptor with MRS1220 significantly reduced the decrease in the number of cells elicited by RSV treatment (Figure 2B) but was unable to modify the reduction in cell viability elicited by RSV (Figures 2A,C).

**FIGURE 3:**
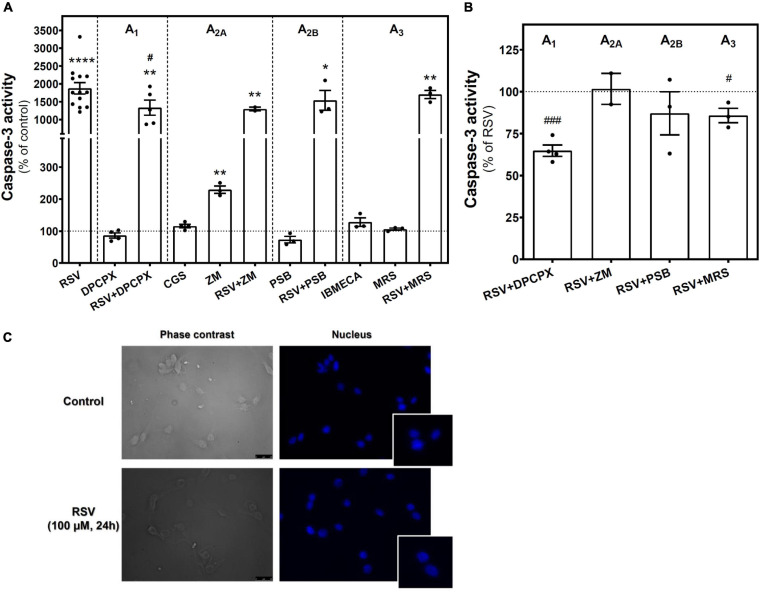
Caspase-3 activity in C6 glioma cells. **(A)** Caspase-3 activity after 24 h of treatment with 100 μM RSV, 10 μM DPCPX, 10 μM CGS, 100 μM ZM, 100 μM PSB, 10 μM IBMECA, and 10 μM MRS alone or in combination with RSV. **(B)** Effect of adenosine receptor antagonists on the activation of caspase-3 elicited by RSV. **(C)** Absence of apoptotic bodies in control and RSV-treated conditions after Hoechst staining of nucleus. Data are means ± SEM of 3–17 independent experiments. *p < 0.05, **p < 0.01 and ****p < 0.0001 significantly different from control condition according to Student’s *t*-test. #*p* < 0.05 and ###*p* < 0.001 significantly different from RSV condition. RSV, resveratrol; DPCPX, 8-Cyclopentyl-1,3-dipropylxanthine; CGS, CGS21680; IBMECA, 2-Cl-IB-MECA; MRS, MRS1220; PSB, PSB1115; ZM, ZM241385.

To assess whether the reduction in cell viability elicited by RSV or other treatments was associated with the induction of apoptosis, the caspase-3 activity was measured. As Figure 3A shows, a strong and significant increase in caspase-3 activity was detected in RSV-treated cells when compared with controls, suggesting the induction of apoptosis by RSV. Caspase-3 activity was also increased after selective blockade of A_2A_ receptor with ZM241385, but more modestly as compared with RSV. Interestingly, blockade of the A_1_ receptor with DPCPX or, to a lesser extent, of the A_3_ receptor with MRS1220 during RSV treatment resulted in lower caspase-3 activation than achieved with RSV alone (Figure 3B). However, no apoptotic bodies were found in stained nuclei after RSV treatment (Figure 3C).

**FIGURE 4:**
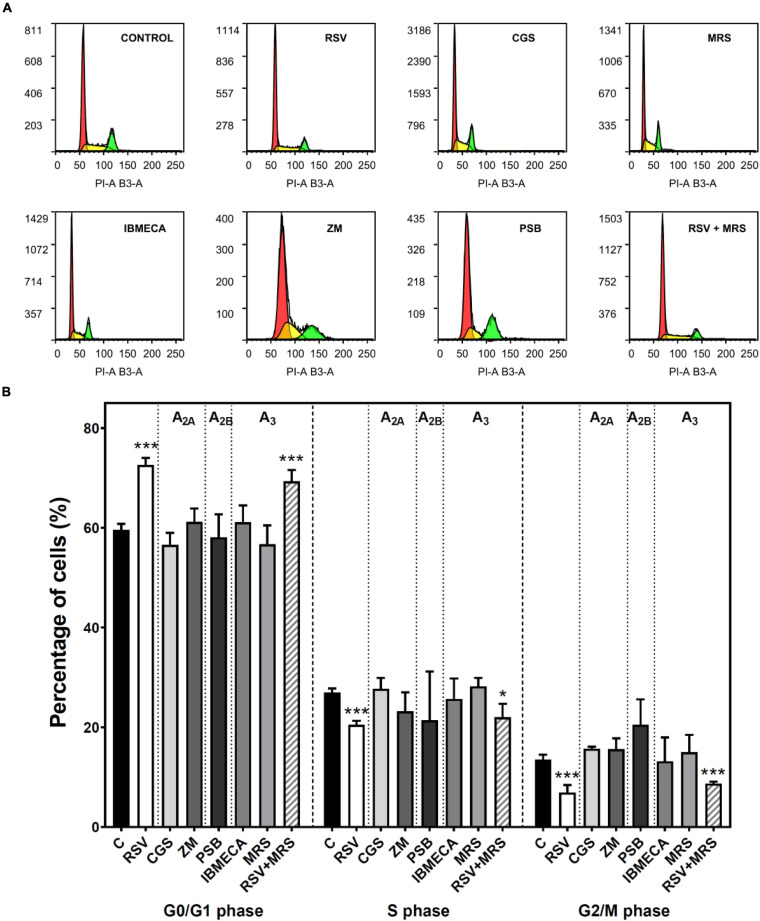
Cell cycle analysis in C6 glioma cells. Cells were analyzed by flow cytometry after 24 h of treatment with 100 μM RSV, 10 μM CGS, 10 μM MRS, 10 μM IBMECA, 100 μM ZM, or 100 μM PSB. **(A)** Representative histogram of cell cycle analysis performed with propidium iodide staining of DNA showing the number of cells versus DNA content (PI-A B3-A). **(B)** Percentage of cells in each cell cycle phase. Data are means ± SEM of 3–15 independent experiments. *p < 0.05 and ***p < 0.001 significantly different from the control condition according to Student’s *t*-test. RSV, resveratrol; CGS, CGS21680; IBMECA, 2-Cl-IB-MECA; MRS, MRS1220; PSB, PSB1115; ZM, ZM241385.

The cell cycle of C6 cells was analyzed after treatment with agonists and antagonists of A_2A_, A_2B_, and A_3_ receptors. The histograms of DNA content obtained by propidium iodide staining and flow cytometry (Figure 4A) were used to calculate the percentage of cells in each cell cycle phase (Figure 4B). This analysis reveals an accumulation of C6 glioma cells in the G_1_ phase after RSV exposure when compared to control conditions, whereas the percentage of cells in the S and G_2_/M phases was significantly diminished, suggesting that cellular division was inhibited. Neither pharmacological blockade nor activation of A_2A_, A_2B_, and A_3_ receptors altered the cell cycle when compared with the control condition, suggesting that RSV action on the C6 cell cycle could not be fully mimicked by selectively targeting these receptors. In addition, no differences were observed when the effect of RSV was analyzed in the presence of MRS1220.

### RSV Effect on Adenosine Metabolism

**FIGURE 5:**
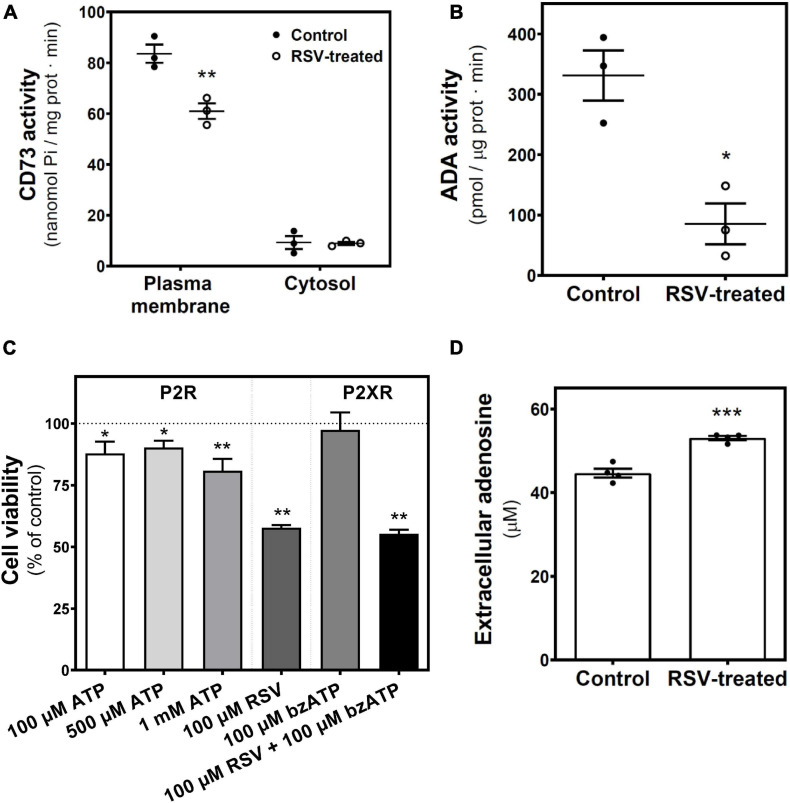
RSV treatment effect on adenosine-related enzymatic activities. 5′-nucleotidase (CD73) and ADA activities were measured in control and 24 h RSV-treated C6 cells. **(A)** 5′-nucleotidase activity localized in the plasma membrane and cytosolic fraction were assayed and represented as nmol Pi/mg prot ⋅ min. **(B)** ADA activity was quantified and represented as pmol/μg prot ⋅ min. **(C)** Cell viability based on the XTT method was performed after 24 h of treatment with the indicated ligands. **(D)** Quantification of adenosine levels in culture medium by HPLC. Data are means ± SEM of three to five independent experiments. *p < 0.05, **p < 0.01, and ***p < 0.001 significantly different from the control condition according to Student’s *t*-test.

Next, we analyzed the enzymatic machinery involved in adenosine production and degradation. We found a significant reduction in 5′-nucleotidase (5′NT or CD73) activity, which catalyzes adenosine synthesis from ATP, localized in the plasma membrane, whereas no changes were observed in the cytosolic fraction (Figure 5A). Likewise, ADA activity, which catalyzes the degradation of adenosine to inosine, was lower in homogenates from RSV-treated cells than in controls (Figure 5B). As the decrease of CD73 activity could lead to the accumulation of ATP metabolites that could stimulate P2X receptors, cell viability was assayed after 24 h treatment with ATP as a non-selective P2R agonist and BzATP as P2XR agonist. Results show a slight but significant decrease in cell viability elicited by high concentrations of ATP that were unable to mimic the RSV effect. BzATP did not change cell viability. Moreover, the RSV effect was maintained in the presence of this P2X agonist (Figure 5C), In addition, extracellular adenosine levels were significantly increased after RSV treatment (Figure 5D).

**FIGURE 6:**
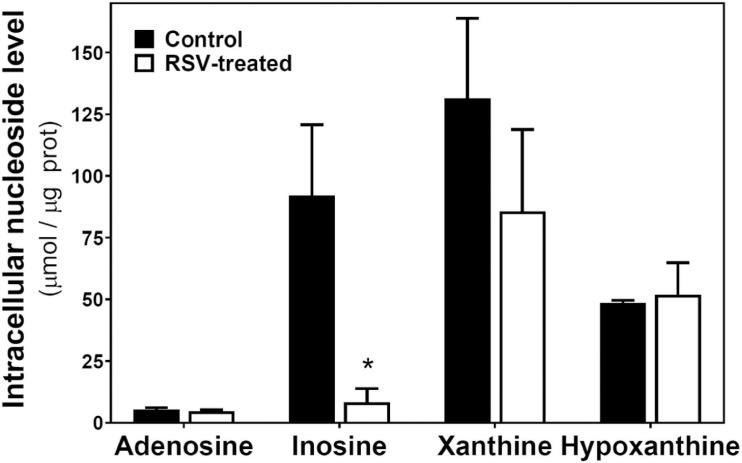
RSV treatment effect on intracellular levels of adenosine and related metabolites in C6 glioma cells. Cells were exposed to RSV at 100 μM for 24 h and adenosine, inosine, xanthine, and hypoxanthine were quantified by HPLC as described in “Materials and Methods.” Data are means ± SEM of three to four independent assays. *p < 0.05 significantly different from the control condition according to Student’s *t*-test.

On the other hand, intracellular levels of adenosine, inosine, xanthine, and hypoxanthine were also quantified (Figure 6). Only inosine levels were strongly and significantly reduced by RSV treatment.

### Adenosine Effect on C6 Glioma Cells Growth

**FIGURE 7:**
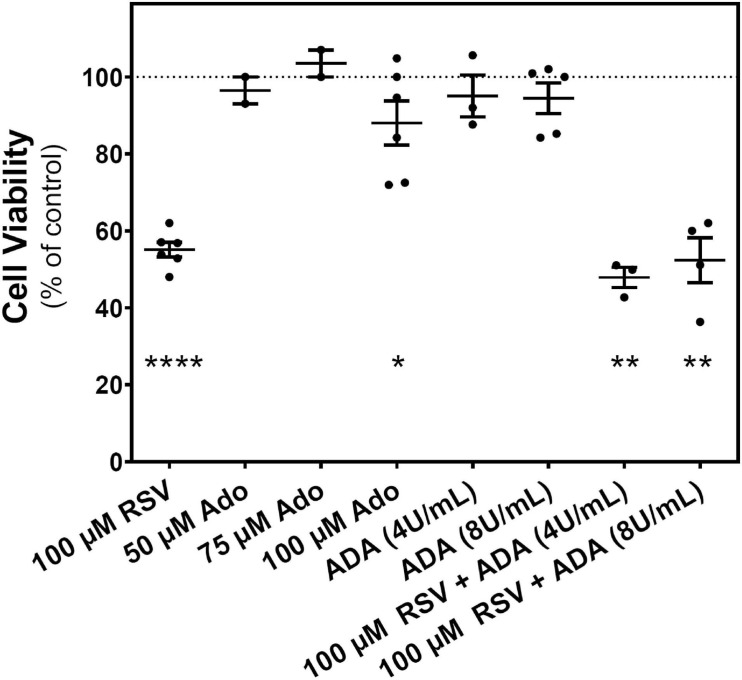
Effect of adenosine on C6 glioma cells growth. Cell viability was measured on C6 glioma cells after 24 h of treatment with 100 μM RSV; 50, 75, and 100 μM adenosine (Ado); 4 or 8 U/mL ADA; or a combination (ADA + RSV). Data are means ± SEM of three to six independent experiments. *p < 0.05, **p < 0.01, and ****p < 0.0001 significantly different from the control condition according to Student’s *t*-test.

After confirming that the extracellular adenosine level was increased by RSV treatment, it was analyzed whether this increase has a role in the reduction of C6 cell growth promoted by RSV. Figure 7 shows that adenosine is unable to mimic the RSV effect on cell viability. Even at 100 μM adenosine, the inhibition of cell viability is more discrete than that observed after RSV treatment. Moreover, the removal of adenosine from the culture medium with ADA, at two different concentrations (4 and 8 U/mL) that ensure the adenosine breakdown during RSV treatment, did not modify the RSV effect on cell viability. As the effect of RSV on cell viability could not be reversed by ADA, direct activation of adenosine receptors by the increased levels of adenosine could be discarded. Instead, a direct action of RSV in adenosine receptors, mainly the A_1_ subtype, could contribute to the antiproliferative effect of RSV.

### Associations Between Components of the Adenosinergic System

**FIGURE 8:**
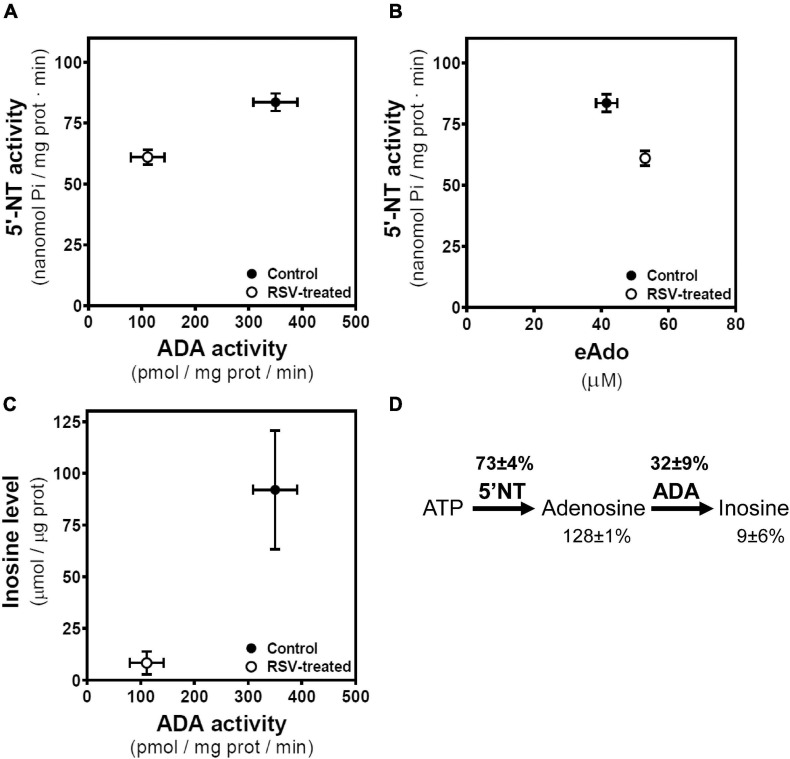
Associations between enzymatic activities and related metabolites. **(A)** Mean values with SEM bars of 5′-nucleotidase (5′-NT) and ADA activities derived from control and RSV treated cells. **(B)** Mean values with SEM bars of 5′-nucleotidase (5′-NT) and extracellular adenosine (eAdo) levels derived from control and RSV-treated cells. **(C)** Mean values with SEM bars of inosine levels and ADA activity derived from control and RSV treated cells. **(D)** Levels of adenosine and inosine and 5′NT and ADA activities in RSV treated cells are expressed as the percentage of the corresponding control value.

Considering the link between levels of adenosine and related metabolites and their enzymatic machinery, we compared their mean ± SEM values obtained in control and RSV-treated cells. In this sense, both 5′NT and ADA activities are reduced by RSV treatment, achieving activities of 73% ± 4% and 32% ± 9% of control values, respectively (Figure 8A). RSV treatment leads to higher levels of extracellular adenosine that seem not to be attributed to higher 5′NT activity, which is reduced by RSV treatment (Figure 8B). Thus, lower inosine levels seem to be associated with lower ADA activity (Figure 8C) in RSV-treated cells. Therefore, the decrease in 5′NT and ADA activities after RSV treatment results in lower inosine (9 ± 6% of control cells) and higher adenosine levels (128 ± 1% of control cells) (Figure 8D).

### Effect of the Inhibition of Phosphodiesterase on C6 Glioma Cells Growth

**FIGURE 9:**
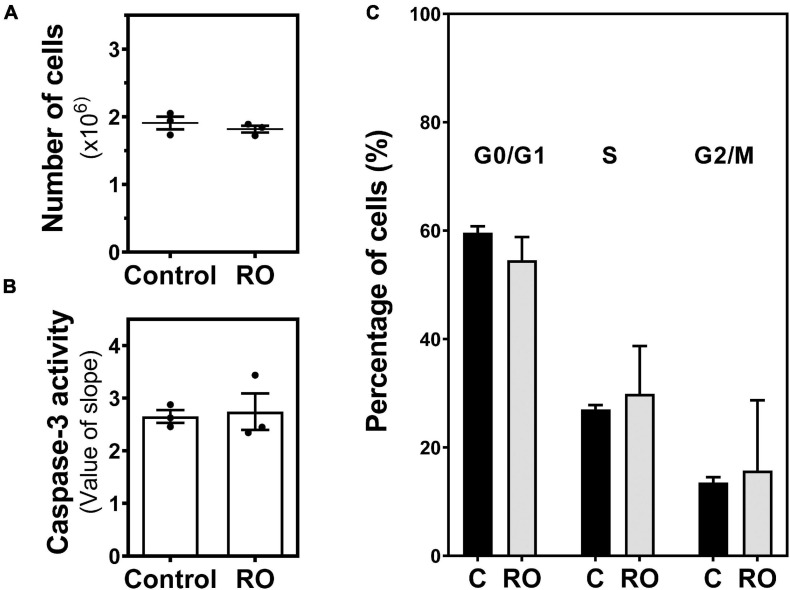
Effect of PDE inhibition on C6 glioma cell growth. Cells were exposed to 100 μM RO-20-1724 (RO) for 24 h and **(A)** the number of cells, **(B)** caspase-3 activity, and **(C)** cell cycle phases were analyzed and measured as described in “Materials and Methods.” Data are means ± SEM of three independent assays.

It is reported that elevation of intracellular cAMP levels through inhibition of phosphodiesterase (PDE) positively correlates with reduced cell proliferation of C6 glioma cells. Therefore, we treated C6 glioma cells with 100 μM RO-20-1724, a selective PDE-IV inhibitor. Our results show that PDE-IV inhibition did not cause a change in the number of cells (Figure 9A), the caspase-3 activity (Figure 9B), or the cell cycle (Figure 9C).

## Discussion

Results presented herein indicate that exposure of C6 glioma cells to RSV caused cell growth inhibition in a time- and concentration-dependent manner by accumulating cells in the G_1_ phase. Caspase-3 activity was increased after treatment. Furthermore, adenosine-converting enzyme activities (i.e., CD73 and ADA) were significantly reduced in RSV-treated cells. In agreement, increased levels of extracellular adenosine were detected, whereas intracellular adenosine remained unaltered. Interestingly, adenosine A_1_ and A_3_ receptors seem to contribute in part to the antiproliferative effect of RSV because the blockade of these receptors partially ameliorates the effect of RSV.

Resveratrol is attracting attention in the prevention of several diseases, including cancer (Jang et al., 1997). However, the precise molecular mechanisms behind its antiproliferative action remain to be clarified. Our results show that RSV induced reduction in cell viability in a time- and concentration-dependent manner and a higher activity of caspase-3, an early apoptosis marker. The absence of apoptotic bodies in the nuclei and a cell cycle arrest in the G_1_ phase, together with a lower percentage of cells in the S and G_2_/M phases, suggests an antiproliferative effect of RSV through cell cycle arrest. A limitation of the cell cycle analysis we performed is that only a single parameter was used for DNA content analysis, and it would be interesting to use an additional specific marker to distinguish between the cell cycle phase. Anyway, we found an IC_50_ value of 78.0 μM (95% CI: 33.2–167.4) for 24 h RSV treatment, very similar to the 85.26 ± 2.14 μM previously reported in C6 cells by Zielinska-Przyjemska et al. (2017). However, these authors report a cell cycle arrest in the S phase after 100 μM RSV exposure for 24 h. Cycle arrest in the S phase is also reported after 100 μM RSV exposure for 48 h (Wang et al., 2015) or 210 μM RSV for 24 h (Zhang et al., 2007). In agreement with our results, it is reported that 100 μM RSV induced cell cycle arrest in the G_1_ phase in breast cancer cells after 24 h but not 48 h treatment (Medina-Aguilar et al., 2016) and in human melanoma cells (Wu et al., 2015) after 48 h. Thus, RSV inhibition of cell cycle progression seems to be cell line specific.

Purinergic signaling is involved in cancer cell proliferation (Di Virgilio and Adinolfi, 2017), and both P2 (Di Virgilio et al., 2018) and P1 (Allard D. et al., 2017) receptors as well as CD39 and CD73 enzymes (Allard B. et al., 2017) could be new targets in cancer. The role of adenosine signaling in cancer is still under debate although it is well accepted that adenosine can promote cancer cell proliferation in several tumors through its receptors (Ohta, 2016; Kazemi et al., 2018). It is reported that adenosine A_2B_ receptors (Sepulveda et al., 2016) and CD73 (Zhang, 2012) are overexpressed in many cancer types. Moreover, adenosine has been found at higher levels in the tumor microenvironment when compared with normal tissue (Ohta et al., 2006) even at a range of 50–100 μM (Vaupel and Mayer, 2016). This tumor-derived adenosine seems to promote cancer cell growth in a receptor-dependent manner as reviewed elsewhere (Ohta, 2016), but it also facilitates immune escape by activating A_2A_ receptors in T cells (Ohta et al., 2006), suggesting a protumor effect of adenosine. However, other authors report a cytotoxic action of adenosine in human cervical cancer cells (Mello Pde et al., 2014), indicating that adenosine might exert a differential action depending on the type of cancer. This cytotoxic effect was also observed in our study when C6 cells were treated with 100 μM adenosine for 24 h. In contrast, it is reported that 100 μM adenosine increased cell proliferation by 36% in U138MG glioma cells (Bavaresco et al., 2008), which might indicate that adenosine action depends not only on the type of cancer (e.g., glioma) but also on the cancer cell line.

Despite the well-known antitumor effect of A_2A_ receptor depletion or pharmacologic inhibition by enhancing the antitumor immune response in mice (Ohta et al., 2006), the specific role of adenosine receptors in the tumor itself remains under debate (Gessi et al., 2011; Di Virgilio and Adinolfi, 2017; Borea et al., 2018; Gorain et al., 2019). Adenosine receptors could display an important action on cancer cell growth, invasion, angiogenesis, and even metastasis (Ohta, 2016; Kazemi et al., 2018). Our work reveals that prolonged pharmacologic blockade of A_2A_ receptors with ZM241385 results in a discrete but significant reduction in the cell viability in conjunction with higher caspase-3 activity, suggesting an antiproliferative effect in cancer cells for A_2A_ receptor antagonists. Other authors report similar data in lung adenocarcinoma tumor cells (Mediavilla-Varela et al., 2013). In a previous study, our group discovered that RSV binds and acts as a non-selective adenosine receptor agonist in C6 glioma cells and that acute RSV treatment altered the A_2A_ receptor/Gs-protein coupling, leading to the inhibition of the cAMP generation upon pharmacologic stimulation of the A_2A_ receptor with CGS21680. Moreover, adenylyl cyclase (AC), PKA protein levels, and basal AC activity were significantly increased after 100 μM RSV treatment for 24 h (Sanchez-Melgar et al., 2019). This dramatic alteration of A_2A_ receptor signaling after RSV treatment makes it difficult to analyze the possible contribution of these receptors to the antiproliferative effect of RSV at least by combining RSV with agonists or antagonists for A_2A_ receptors as employed here. Diet supplementation with RSV also caused the desensitization of A_2A_ receptors in the brain from SAMP8 mice (Sanchez-Melgar et al., 2018). These results might support that the alteration of A_2A_ receptor signaling could be involved in the antiproliferative action of RSV. It is reported that elevation of intracellular cAMP levels through either activation of AC or inhibition of PDEs leads to PKA activation and positively correlates with reduced cell proliferation of C6 glioma cells. Interestingly, the elevation of cAMP levels with forskolin induces cell cycle arrest of C6 glioma cells in the G_2_/M phase. In comparison, inhibition of PDEs not only inhibits cell growth *via* the cAMP/PKA cascade, but also triggers cell death through caspase-3/-7 activation (Safitri et al., 2020). It is described that anticancer agents, such as RSV, may act by modulating cell cycle–associated proteins, such as cyclins, cyclin-dependent kinase (CDK), and CDK inhibitors (Wolter et al., 2001). CDK inhibitors are shown to be the downstream targets of caspase-3 activation, and loss of these inhibitors can result in the aberrant upregulation of CDKs that have been associated with apoptotic cell death (Jin et al., 2000). Therefore, RSV-induced G_1_-phase cell cycle arrest could be mediated through the caspase/cyclin-CDK pathways. In agreement, the protein content of the cycle arrest proteins CDK2, CDK4, cyclin D1, PCNA, and P21 is reported to be decreased in a concentration-dependent manner in RSV-treated (100 μM, 24 h) HCT116 and Caco-2 cells compared with control cells (Liu et al., 2014). Moreover, RSV inhibits human U251 glioma cell proliferation and induces G_0_/G_1_ growth arrest, and these effects are reduced by the CDK inhibitor olomoucine (Jiang et al., 2005). These RSV effects on cell cycle and viability *via* caspase-3 activation could be modulated by different adenosine receptors after their activation by binding of adenosine or even RSV. In fact, treatment of C6 glioma cells with 25 μM Cl-IB-MECA reduced Bcl-2 expression and increased caspase-3 activity after 24 h of treatment. This apoptotic effect was observed only with activation of the A_3_ receptor, whereas activation of the A_1_ or A_2A_ receptors did not induce significant apoptotic effects (Appel et al., 2001). However, activation of A_1_ receptors with CPA increased the cell viability and reduced apoptosis, and the antagonist DPCPX significantly induced apoptosis and caspase-3 expression in MCF-7 cells (Dastjerdi et al., 2016).

Among the molecular targets of RSV reported to date, the AMP-activated protein kinase (AMPK) can be found (Kulkarni and Canto, 2015). The activation of AMPK is reported to suppress the proliferation of various cancers *via* the regulation of cell cycle progression, apoptosis, autophagy, inhibition of protein synthesis, and *de novo* fatty acid synthesis. AMPK causes G_1_ cell cycle arrest *via* upregulation of the tumor suppressor protein p53, which upregulates p21, a CDK inhibitor (Motoshima et al., 2006). AMPK is a trimer with α-, β-, and γ-subunits. The α-subunit contains the kinase domain, and its Thr^172^ residue is phosphorylated (p-AMPK) by an upstream kinase and determines its regulation. The binding of AMP and, to a lesser extent, ADP to the γ-subunit stimulates AMPK activity. Thus, changes in the ATP/ADP or ATP/AMP ratio lead to the allosteric activation of AMPK (Herzig and Shaw, 2018). Therefore, enzymes involved in the modulation of AMP levels due to the conversion of AMP to adenosine (i.e., 5′NT) or adenosine to AMP (i.e., adenosine kinase) could control AMPK activation. Extracellular adenosine activates AMPK (Aymerich et al., 2006); however, PKA-mediated inhibition of AMPK *via* increased inhibitory phosphorylation of AMPK^*Ser173*^ and reduced activating phosphorylation of AMPK^*Thr172*^ is reported (Djouder et al., 2010; Aw et al., 2014). This PKA-mediated inhibition of AMPK could take place in C6 glioma cells as cAMP/PKA signaling is significantly increased, and the AMPK activation (measured as the p-AMPK/AMPK ratio) is not modified in these cells after 100 μM RSV treatment for 24 h (Sanchez-Melgar et al., 2019). Therefore, the cell cycle arrest elicited by RSV in C6 glioma cells seems to be independent of the AMPK/p53/p21 mediated inhibition of CDKs.

The enzyme CD73 has gained attention since it was discovered that adenosine is one of the major constituents in the tumor microenvironment (Di Virgilio and Adinolfi, 2017) and that this tumor-derived adenosine facilitates the immune escape by activating the A_2A_ receptor in T and NK cells (Ohta and Sitkovsky, 2014). CD73 overexpression in tumor cells is associated with the pathogenesis (Yan et al., 2019), progression (Yu et al., 2018), and poor prognosis in several types of cancers, including HNSCC (Ren et al., 2016a), triple-negative breast cancer (Loi et al., 2013), oral squamous cell carcinoma (Ren et al., 2016b), and high-grade serous ovarian cancer (Turcotte et al., 2015), among others. Therefore, targeting CD73 with selective inhibitors or antibodies is being considered as a promising therapeutic strategy against cancer (Stagg et al., 2010). Our data indicate a significant reduction in the CD73 activity located in the plasma membrane fraction after RSV exposure, suggesting that RSV might affect adenosine production in the pericellular space. Nevertheless, a lower enzymatic activity of ADA was also found after RSV treatment, leading to lower deamination of adenosine into inosine and significantly decreased inosine levels. A limitation of the technique employed here to measure CD73 activity could be that we cannot rule out the contribution of alkaline phosphatase in the conversion of AMP to adenosine (Zimmermann, 2021). However, the presence of 100 μM levamisole, a selective alkaline phosphatase inhibitor, during the assay determining CD73 activity in the human cerebral cortex modified the activity of CD73 in neither membranes nor cytosolic fraction by using the same assay conditions as here (Alonso-Andres et al., 2018). This reduced CD73 activity could promote an increased level of ATP. It is well known that extracellular ATP may exhibit a cytotoxic effect in cancer cells depending on the concentration (Vultaggio-Poma et al., 2020). Among P2 receptors, the P2 × 7 receptor subtype seems to be the main player in ATP-dependent biological actions. Prolonged activation of P2 × 7, *via* high levels of extracellular ATP over an extended time period, can lead to the formation of a macropore, leading to depolarization of the plasma membrane and, ultimately, to cell death (Lara et al., 2020). However, our results show that, after 24 h of P2X stimulation with BzATP, C6 cell viability did not change, and this prolonged stimulation was unable to modify the RSV effect.

A highly active CD73 enzyme has been detected in glioblastoma (Ludwig et al., 1999) and glioma (Bavaresco et al., 2008). Independently of its enzymatic role, CD73 can mediate cell–cell adhesion being a coreceptor in T cell activation or regulate cell interaction with ECM components and migration on them. Acting as a docking molecule, CD73 mediates migration and invasion of A375 melanoma cells (Sadej and Skladanowski, 2012) and glioblastoma invasiveness (Fenoglio et al., 1997) through focal adhesion kinase activation. Interestingly, RSV was able to regulate the invasion of cancer cells by modulating such focal adhesion kinase (Buhrmann et al., 2017), which could be mediated by the RSV effect on the CD73 enzyme.

The precise molecular mechanism by which RSV modulates CD73 activity is not clarified yet. It is described a possible link between hypoxia-inducible factor-1 alpha (HIF-1α) and CD73 (Sotnikov and Louis, 2010). Hypoxic conditions in the tumor trigger HIF-1α activation and, in turn, an upregulation of the CD73-adenosine pathway (Li et al., 2017), which is able to promote tumor growth and metastasis (Zhang, 2012). RSV treatment reduced HIF-1α in cancer cells *in vitro* (Zhang et al., 2005), which might explain the reduction in the CD73 activity observed in RSV-treated cells in our study. Of interest, moderate hypoxia (24 h at 5% O_2_) produces increased endogenous adenosine levels in C6 glioma cells and the downregulation and upregulation of A_1_ and A_2A_ receptors, respectively. However, HIF-1α was not modulated by moderate hypoxia, and C6 cells were resistant to cell death elicited by hypoxic insult (Castillo et al., 2008).

A growing body of evidence indicates that dual blockade of CD73 and A_2A_ receptors could enhance the antitumor response (Beavis et al., 2015; Young et al., 2016). In this sense, RSV induces the reduction in CD73 activity as observed in our study and changes the A_2A_ receptor downstream signaling from activation to inhibition of adenylyl cyclase as we reported previously (Sanchez-Melgar et al., 2019).

Reduced CD73 and ADA activities as reported herein result in increased extracellular levels of adenosine after RSV exposure. However, RSV-induced cell growth inhibition seems to be independent of the activation of adenosine receptors by extracellular adenosine because of (1) an adenosine concentration of 50 μM, similar to that achieved after RSV treatment, and 75 μM were unable to reduce cell viability, (2) 100 μM adenosine treatment slightly decreased cell viability but to a lesser extent than observed with RSV treatment, and (3) ADA presence during RSV treatment did not impede the RSV effect on these cells. Instead, direct activation of adenosine receptors by RSV itself acting as a non-selective agonist seems to be involved as we recently suggested (Sanchez-Melgar et al., 2019). Pharmacological inhibition of the A_1_ receptor with DPCPX or the A_3_ receptor with MRS1220 during RSV treatment resulted in a significantly reduced RSV effect, suggesting possible participation of reduced levels of cAMP after RSV binding to these Gi-protein coupled receptors (i.e., A_1_ and A_3_). Moreover, prolonged pharmacologic inhibition of the A_2A_ receptor with ZM241385 or the A_2B_ receptor with PSB1115 partially mimicked the RSV-induced effect on C6 cell viability. As mentioned, RSV treatment altered the A_2A_ receptor/Gs-protein coupling, leading to the inhibition of the cAMP generation upon pharmacologic stimulation of the A_2A_ receptor with CGS21680 after RSV treatment (Sanchez-Melgar et al., 2019). All these data might indicate that the reduction of C6 cell growth upon RSV treatment could be related to the inhibition of cAMP levels through adenosine receptor modulation, mainly A_1_ and A_3_ receptors.

**FIGURE 10:**
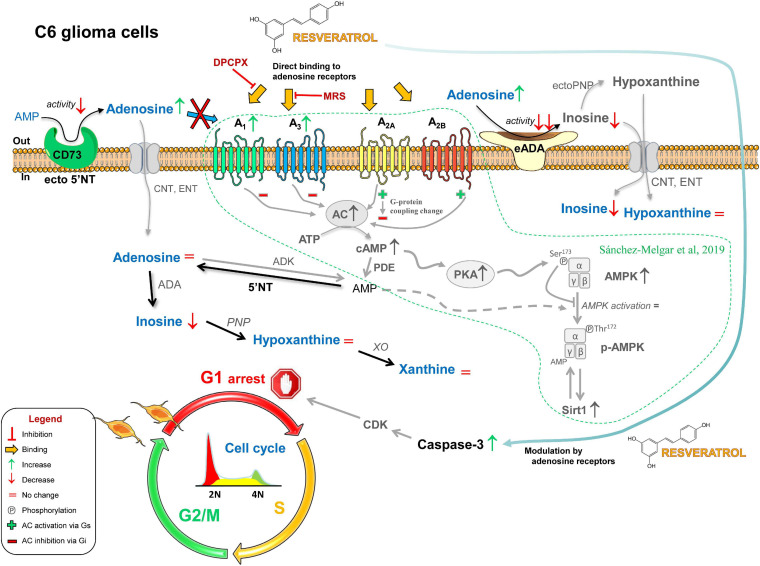
Possible role of adenosinergic signaling in the antitumoral effect of RSV. The exposure of C6 glioma cells to RSV causes the decrease (↓) or the increase (↑) of several compounds and proteins that ultimately lead to cell cycle arrest in the G_1_ phase. RSV upregulates A_1_ and A_3_ receptors; changes G-protein coupling of A_2A_ receptor from activation (Gs, +) to inhibition (Gi, −) of adenylate cyclase (AC) activity; upregulates AC, increases basal AC activity, and upregulates protein kinase A (PKA); higher levels of cAMP inhibit AMPK activation *via* PKA-mediated phosphorylation (Ser^172^); reduces adenosine-converting enzymes (i.e., CD73 and ADA), leading to increased extracellular levels of adenosine; caspase-3 is activated as a result of the direct binding of RSV to adenosine receptors and the modulatory action of these receptors on other RSV-elicited pathways, leading to CDK-mediated cell cycle arrest in the G_1_ phase. Previous results reported by our group in these cells are shown in gray and surrounded by a dotted line.

In summary, our study suggests that a reduced CD73 activity located in the plasma membrane in addition to a fine-tuned modulatory role of adenosine receptors could be involved, at least in part, in the antiproliferative action of RSV in C6 glioma cells (Figure 10).

## Data Availability Statement

The original contributions presented in the study are included in the article/Supplementary Material, further inquiries can be directed to the corresponding author/s.

## Author Contributions

MM and JA: conceptualization, writing—review and editing. AS-M, MM, and JA: formal analysis. MM: funding acquisition. AS-M and SM-L: investigation. AS-M and MM: writing—original draft. All authors have read and agreed to the published version of the manuscript.

## Conflict of Interest

The authors declare that the research was conducted in the absence of any commercial or financial relationships that could be construed as a potential conflict of interest.

## Publisher’s Note

All claims expressed in this article are solely those of the authors and do not necessarily represent those of their affiliated organizations, or those of the publisher, the editors and the reviewers. Any product that may be evaluated in this article, or claim that may be made by its manufacturer, is not guaranteed or endorsed by the publisher.

## Supplementary Material

Supplementary Video 1 Growth of C6 glioma cells in control conditions or in the presence of 0.1–100 μM RSV. Time-lapse movie (one frame/2 min) of cells growing for 24 h since the start of treatment. Bar size: 100 μm.
