# Supplementary figures and images for: Antitumoral Action of Resveratrol Through Adenosinergic Signaling in C6 Glioma Cells
Source: Front Neurosci. 2021 Sep 1;15:702817. doi: 10.3389/fnins.2021.702817 (PMC8440868; doi:10.3389/fnins.2021.702817)

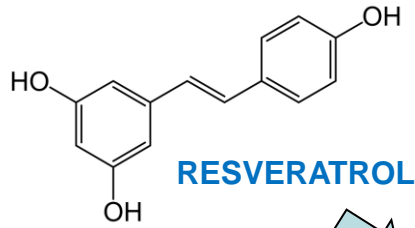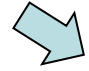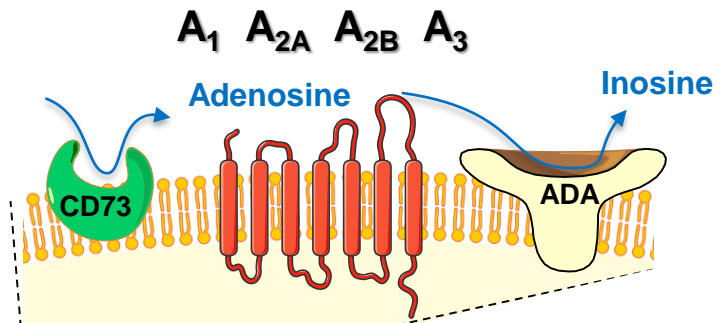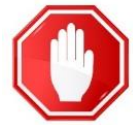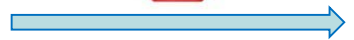

**Cell cycle arrest**

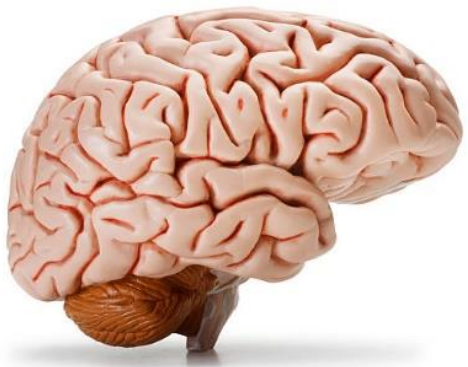

**C6 glioma cells**

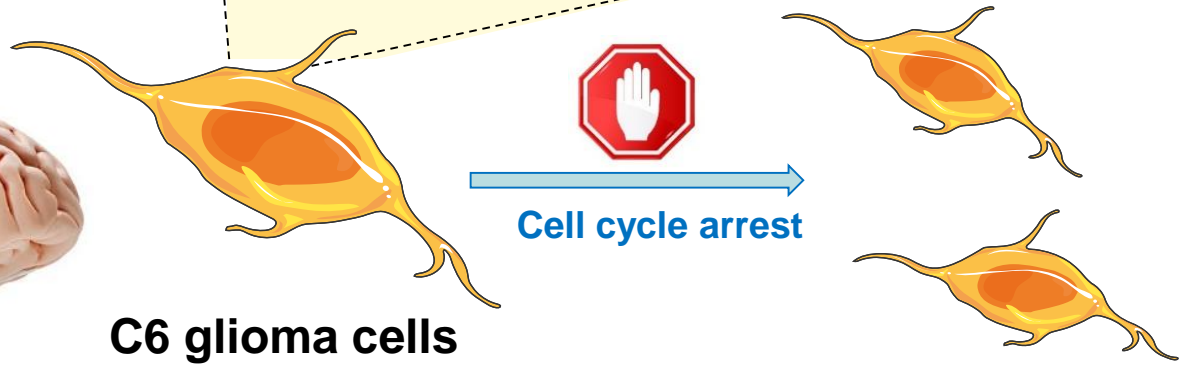

Supplement: Supplementary file 2 [file Presentation_1.PDF]
